# Potential of animal-welfare compliant and sustainably sourced serum from pig slaughter blood

**DOI:** 10.1007/s00441-024-03904-8

**Published:** 2024-07-11

**Authors:** Olga Hahn, Kirsten Peters, Alexander Hartmann, Dirk Dannenberger, Claudia Kalbe

**Affiliations:** 1 Institute for Cell Biology, University Medical Center Rostock, Rostock, Germany; 2 Institute of Clinical Chemistry and Laboratory Medicine, University Medical Center Rostock, Rostock, Germany; 3 Research Institute for Farm Animal Biology (FBN), Wilhelm-Stahl-Allee 2, D-18196 Dummerstorf, Germany

**Keywords:** FBS replace, Adult stem cells, Human, Proliferation, Differentiation

## Abstract

The animal product most used as a stimulatory additive for cell cultivation is still fetal bovine serum (FBS). Besides the ethical concerns regarding serum collection, the main problems of FBS are batch-to-batch variability and the resulting risk of lower reproducibility, the differences between species, the presence of undefined/unknown components, and the risk of contamination. In contrast, pig blood, which is a by-product of slaughter, is a sufficiently available and sustainable resource with a high degree of standardization in terms of donor age, weight, and genetics. The variations in preparations from pig slaughter blood seem to be comparatively low, and consequently, batch effects might be much smaller, suggesting that the reproducibility of the research data obtained may be increased. Our pilot study aimed to investigate, as a proof of concept, whether adult human and porcine stem cells of different tissue origins proliferate and differentiate adequately when FBS is completely or partially replaced by porcine serum (PS). We could show that the human and porcine stem cells were vital and proliferated under partial and full PS supplementation. Furthermore, using PS, the two cell types studied showed tissue-specific differentiation (i.e., lipid vacuoles as a sign of adipogenic or myotubes as a sign of myogenic differentiation). In conclusion, the pig slaughter blood–derived serum has promising potential to be a replacement for FBS in adult stem cell cultures. Therefore, it could serve as a basis for the development of new cell culture supplements.

**Supplementary Information:**

The online version contains supplementary material available at 10.1007/s00441-024-03904-8.

## Introduction

The global use of fetal bovine serum (FBS) in in vitro cell culture model systems results in an annual production of about 800,000 l, derived from about 2 million fetuses (Brindley et al. 2012). Besides the fundamental ethical concerns with the use of FBS, the main problems identified are intra-batch variability resulting in low reproducibility, the presence of undefined/unknown components, and the risk of contamination (Cassotta et al. 2022; van der Valk 2022). Therefore, FBS-free media are considered the solution of the future in cell cultures. However, the currently commercially available, chemically defined serum substitutes require a very time-consuming and cost-intensive conversion of the cell culture model systems and do not always lead to the desired cell characteristics (Kolkmann et al. 2020). In addition, the use of cell culture supplements of non-animal origin is currently associated with problems, such as the limited availability of already standardized and established protocols and the high costs. Thus, FBS remains the most commonly used animal product as a stimulatory additive for the cultivation of primary cells and cell lines (Cassotta et al. 2022).

Pig blood can be obtained as a by-product of meat production during the welfare-conscious slaughter of stunned pigs and is therefore a sustainable resource. Slaughter pigs have a high degree of standardization in terms of age, weight, and genetics, which means that variation in their whole-blood preparations is comparatively low, and consequently, there should be lower batch effects. This could lead to a higher data reproducibility than in the FBS-based in vitro models and thus to a reduction of animal experiments in the sense of the 3R strategy according to Russell and Burch (1959).

To investigate the potential of porcine serum (PS) for FBS replacement, we examined the effects on primary cells, i.e., adult human and porcine stem cells, for their proliferation and differentiation potential. These cell types were chosen because they play an important role in biomedical research (Meyer et al. 2015; Geng et al. 2022). Mesenchymal stem/stromal cells (MSC) are responsible for the maintenance of tissue-typical properties and the regeneration of age- or injury-related tissue. Due to their multipotent differentiation potential, a comparatively simple isolation procedure, and their regenerative and immunomodulatory properties, they are of great interest for cell therapeutic applications (Zuk 2010; Meyer et al. 2015). MSC have already been used as model cells for replacing FBS with human platelet lysates (Rauch et al. 2014a, b). Satellite cells of skeletal muscle tissue (musSC) are adult stem cells that serve the hypertrophic growth and regeneration of skeletal muscle (Mauro 1961). Upon isolation from skeletal muscle tissue, these cells can be used to simulate adult myogenesis with proliferating myoblasts and differentiating myotubes in culture (Metzger et al. 2020). In addition to the essential role in biomedical research (Geng et al. 2022), musSC form the cellular basis for the production of so-called in vitro meat, i.e., meat from the petri dish (Bhat et al. 2019).

In our initial proof-of-concept study, we investigated whether PS from pig slaughter blood induces similar proliferation rates and tissue-specific differentiation in human adipose tissue–derived MSC (adMSC) and porcine musSC (i.e., adipocytes and myotubes) when FBS is completely or partially replaced.

## Materials and methods

### Adult stem cells and experimental approach

Human adipose–derived mesenchymal stem/stromal cells (adMSC) were isolated, cultivated, and cryopreserved in a standardized manner according to the previously described instructions (Meyer et al. 2015; Klemenz et al. 2019; Hahn et al. 2020). Porcine skeletal muscle–derived satellite cells (musSC) were isolated and handled as described by Metzger et al. (2020). Details of human subjects and animals were given in Supplementary information. All experiments were done with cells in passage four. The adMSC are usually cultured with 10% FBS from PAN Biotech (Aidenbach, Germany, Lot No.: P160406) (control adMSC), whereas the musSC are cultured in 10% FBS from Sigma-Aldrich (Saint Louis, USA, BCBW7811) and 10% horse serum (HS; control musSC). Depending on the experimental approach, FBS was completely (10% PS) or partially (5% FBS + 5% PS) replaced by PS without or with heat inactivation.

For serum collection, ten female fattening pigs (171 ± 15 days of age) with a body weight of 124 ± 5 kg were killed using exsanguination after electro stunning. During exsanguination, 200 ml of slaughter blood per animal was sampled. For the coagulation process, tubes were then stored lying down for at least 60 min in the dark at room temperature (RT). This was followed by a centrifugation step for 10 min at RT in a swing-out rotor at 2500 × g without brake. Subsequently, the clear serum from all donors was pooled and sterile filtered twice through a 0.2-µm filter (Filtropur, Sarstedt, Nümbrecht, Germany). Half of the serum thus obtained was stored in a water bath at 56 °C for 30 min for heat inactivation. The heat inactivation procedure was considered here, as the serum was from 171-day-old pigs whose complement system was developed. All sera were stored at − 20 °C until use.

### Sera analyses

Concentrations of serum parameters were analyzed with a clinical chemistry analyzer (Pentra C400, HORIBA ABX SAS, Montpellier, France) using commercial kits (Daş et al. 2016). Serum samples were deproteinized with 1.5 M HClO_4_, neutralized with 2 M K_2_CO_3_ (ratio 3:2:1, v:v:v), and centrifuged at 50,000 × g and 4 °C for 20 min. Glucose and lactate in the supernatant were analyzed by HPLC (Metges et al. 2014) using a Series 1200/1260 Infinity II system (Agilent Technologies, Waldbronn, Germany) with refractive index detection. To determine the concentration of insulin, C-peptide, and vitamin B12, the cobas C 508 for clinical chemistry (Roche, Mannheim, Germany) was used according to the manufacturer’s instructions. The selenium concentration was determined by a medical contract laboratory (www.labor-lademannbogen.de).

Serum-free amino acids and amino metabolites were measured by HPLC after tenfold dilution by ultrapure water and pre-column derivatization, as described earlier (Kuhla et al. 2010). For separation, a Gemini^®^ 250 × 4.6 mm 5 µm C18 110 Å column protected by a 4 × 3 mm pre-column (both Phenomenex, Aschaffenburg, Germany) was used on a Series 1260 Infinity II system (Agilent Technologies, Waldbronn, Germany) with fluorimetric detection.

The fatty acid concentrations in sera were analyzed after total lipid extraction and transesterification of fatty acids to corresponding methyl ester using capillary gas chromatography (GC). The analysis was performed using a CP-Sil 88 CB column (100 m × 0.25 mm, Agilent, Santa Clara, CA, USA) that was installed in a PerkinElmer gas chromatograph CLARUS 680 with a flame ionization detector and split injection (PerkinElmer Instruments, Shelton, USA) as described by Gnott et al. (2020).

The different sera were first centrifuged for 5 min at 10,000 × g for sterility testing. Afterward, the supernatant was cultivated in a Brain-Heart infusion solution, whereas the pellets were cultivated on Columbia agar plates under aerobic and anaerobic conditions for up to 48 h. To determine the endotoxin content of the sera, the Pierce™ chromogenes Endotoxin Quant Kit (ThermoFisher Scientific, Schwerte, Germany) was used according to the manufacturer’s instructions.

### Proliferation capacity

For cell number determination, adMSC were seeded at a density of 6000 cells/cm^2^ in uncoated wells and musSC at a density of 4500 cells/cm^2^ in gelatin-coated dishes in their specific proliferation media described in Supplementary information. After 24 h of recovery, the proliferation media was renewed for the control (adMSC, 10% FBS; musSC, 10% FBS and 10% HS) or changed to 10% PS or 5% FBS and 5% PS for another 72 h. After 72 h of cultivation, cell numbers and viability were analyzed using the NucleoCounter^®^ NC-3000™ Viability and Cell Count Assay (Chemometec, Allerod, Denmark) as endpoint determination according to the manufacturer’s instructions.

Furthermore, for real-time monitoring, adMSC and musSC were seeded in comparable densities as described for cell number determination in e-plates equipped with microelectrode biosensors at the bottom of each well (e-plates 96, ACEA Biosciences, Heidelberg, Germany). As described above, all cells were cultured for 24 h in proliferation media including 10% FBS or 10% FBS and 10% HS for adMSC or musSC, respectively. Thereafter, the media was renewed for the control or changed to the different sera (10% PS or 5% FBS + 5% PS) for an additional 72 h. The adMSC and musSC were used from three donors with three technical replicates. Real-time impedance monitoring assays were performed as previously reported (Metzger et al. 2020). Briefly, impedance was recorded every 30 min, providing normalized cell index (nCI, arbitrary units, normalization after 24 h) values. The cell proliferative growth parameters between 24 and 96 h (72 h of growth) were calculated with the RTCA software 1.2.1 (ACEA BiosciencesA) and included the slope (1/h).

### Differentiation capacity

The adMSC were seeded at a density of 20,000 cells/cm^2^ for the differentiation assays, and cells were cultured for up to 14 days with a specific differentiation medium (Supplementary information). The adipogenic differentiation procedure was performed according to Meyer et al. (2015), Wolff et al. (2022), and Waheed et al. (2022). Briefly, 72 h after seeding (defined as day 0), different sera (10% PS or 5% FBS + 5% PS) were added to the proliferation medium or the adipogenic differentiation medium. The adMSC cultured with 10% FBS served as control cultures. After 14 days of cultivation, adipogenic differentiation was determined by BODIPY staining, and all images for cell count were quantified using a Hoechst H33342 stain (5 µg/ml, Applichem, Darmstadt, Germany) as previously described (Meyer et al. 2015; Waheed et al. 2022; Wolff et al. 2022). Metabolic activity of adMSC was determined using the CellTiter 96 Aqueous One Solution Cell Proliferation Assay (MTS, Promega, Madison, WI, USA) on days 0 and 14 after serum supplementation according to the manufacturer’s instructions.

The musSC were seeded at a density of 6800 cells/cm^2^ in Geltrex™-coated (growth factor reduced, 1:100, Gibco Thermo Fisher Scientific, Dreieich, Germany) culture dishes. The myogenic differentiation procedure was performed according to Metzger et al. (2020). After 24 h of growth in the proliferation medium (Supplementary information), a medium change was performed either with proliferation medium (control) or different sera (10% PS or 5% FBS + 5% PS). At a confluency of 80%, the proliferation medium was changed to proliferation medium 2 (Supplementary information) with 1 µM insulin (Sigma-Aldrich, Saint Louis, MO, USA) and without HS, until the cells were 100% confluent. Further differentiation was usually done with a completely serum-free myogenic differentiation medium (Supplementary information). The estimation of fusion degree was performed after 4 days of cultivation with differentiation medium as described by Mau et al. (2008) defining a myotube as a desmin-positive cell with two or more nuclei.

### Data illustration and statistical analysis

All analyses were performed independently on cells from three different donors (human or porcine) for xCelligence or from five different donors, each in triplicate, and the mean of each triplicate was used for one individual and compared with their controls. Data were visualized and statistically analyzed using Microsoft Excel 2010 (Microsoft, Redmond, WA) and GraphPad Prism, version 7.00 (GraphPad Software Inc., San Diego, CA, USA). Since the data received were distributed normally (Shapiro-Wilk test), the statistical significance within the dataset was calculated using an ordinary one-way analysis of variance (ANOVA) or a two-way ANOVA followed by Dunnett’s multiple comparison post hoc test, with the significance level set at a *P* value of 0.05. The statistical significance between datasets for data which was not normally distributed was calculated with the Kruskal-Wallis test by Dunn’s multiple comparison post with a *P* value of 0.05.

## Results and discussion

### Sera characterization

The adMSC were cultured with the routinely used FBS from PAN Biotech (FBS-PAN), while the musSC were cultured with FBS from Sigma-Aldrich (FBS-Sigma). For both cell types, FBS was replaced by PS, which was obtained from the slaughter blood of 10 female fattening pigs. The results of sera characterization are presented in Table 1, including general parameters (e.g., pH or endotoxin content) that are routinely measured by the FBS-producing companies and made available to users. In addition, carbohydrate glucose, proteins/amino acids, lipid metabolism–associated components, some micronutrients, vitamins, and metabolites were quantified. Detailed data on the amino acid and fatty acid concentrations of the sera are summarized in Supplementary Tables 1 and 2, respectively. The results are consistent with many studies that have shown significant differences for different FBS or FBS batches (van der Valk 2022). As illustrated in Table 1, the vast majority of our PS parameters (e.g., protein content, albumin, or cholesterol) fall within a range that is very similar to that of both commercial FBS. For more than half of the parameters, PS is even between FBS-PAN and FBS-Sigma, for instance, for most amino acids. However, there are greater variations in the higher fatty acid contents of PS compared with FBS, which can be attributed to the feeding of the fattening pigs (Dannenberger et al. 2012). Further research is required to investigate the potential batch effects of PS.

**Table 1:** Basic characterization of the two routinely used fetal bovine sera (FBS) and the sustainable pig serum (PS) obtained from slaughter blood

**Parameter**	**FBS** (PAN Biotech, Lot No. P160406)	**FBS** (Sigma-Aldrich, BCBW7811)	**PS**
**General parameters**			
pH value	7.4	7.6	8.4
Total bilirubin (µmol/l)	6.0	1.5	1.2
Endotoxin^a^ (EU/ml)	25.4	< 0.2	5.5
Sterility	Pass	Pass	Pass
**Carbohydrates**			
Glucose (mmol/l)	11.3	3.3	6.2
**Protein content and amino acids**^**b**^			
Total protein (g/l)	53.6	36.6	58.7
Albumin (g/l)	35.3	22.2	37.3
Arg (arginine, µM)	184.7	32.3	80.4
Cys (cysteine, µM)	63.2	19.9	54.2
Gln (glutamine, µM)	315.5	370.8	331.9
His (histidine, µM)	167.3	71.7	83.6
Ile (isoleucine, µM)	535.6	121.3	132.8
Leu (leucine, µM)	625.2	203.2	244.4
Lys (lysine, µM)	557.2	174.2	238.6
Met (methionine, µM)	122.2	18.6	33.1
Phe (phenylalanine, µM)	324.2	128.8	95.3
Thr (threonine, µM)	536.6	140.4	184.3
Trp (tryptophan, µM)	95.8	46.4	64.0
Tyr (tyrosine, µM)	238.0	85.8	68.6
Val (valine, µM)	679.0	304.6	422.8
**Lipid analyses**^**c**^			
Triglycerides (mmol/l)	0.9	0.7	0.7
NEFA^d^ (µmol/l)	493	59	303
Cholesterol (mmol/l)	2.1	0.8	2.3
HDL^e^ cholesterol (mmol/l)	1.2	0.3	1.1
LDL^f^ cholesterol (mmol/l)	0.6	0.5	1.0
Sum SFA^g^ (µg/g)	324.5	161.0	482.7
Sum MUFA^h^ (µg/g)	215.7	101.6	337.0
Sum PUFA^i^ (µg/g)	371.4	68.6	677.4
Sum n-3 PUFA (µg/g)	163.4	22.9	41.4
Sum n-6 PUFA (µg/g)	201.3	43.4	633.1
**Micronutrients**			
Calcium (mmol/l)	5.6	3.5	2.7
Iron (µmol/l)	48.1	37.1	28.2
Selenium (µmol/l)	0.1	0.1	1.9
**Vitamin and hormone**			
Vitamin B12 (pg/ml)	> 2000	360	343
Insulin (µU/ml)	< 0.4	0.6	0.6
C peptide (nmol/l)	< 0.007	< 0.007	< 0.007
**Metabolites**			
Lactate (mmol/l)	16.9	18.5	6.6
Creatinine (µmol/l)	217.1	280.6	162.6
Uric acid (µmol/l)	126.2	81.9	5.7
Urea (mmol/l)	4.9	5.2	4.2

^a^Endotoxin values for FBS were given by the supplier

^b^Amino acids generally considered essential for cultured cells, a complete overview of all analyzed amino acids is given in Supplementary Table 1

^c^The complete fatty acid profile can be found in Supplementary Table 2

^d^*NEFA* non-esterified fatty acids

^e^*HDL* high-density lipoprotein

^f^*LDL* low-density lipoprotein

^g^*SFA* saturated fatty acids

^h^*MUFA* mono-unsaturated fatty acids

^i^*PUFA* poly-unsaturated fatty acids

### Cell proliferation

**Fig. 1:**
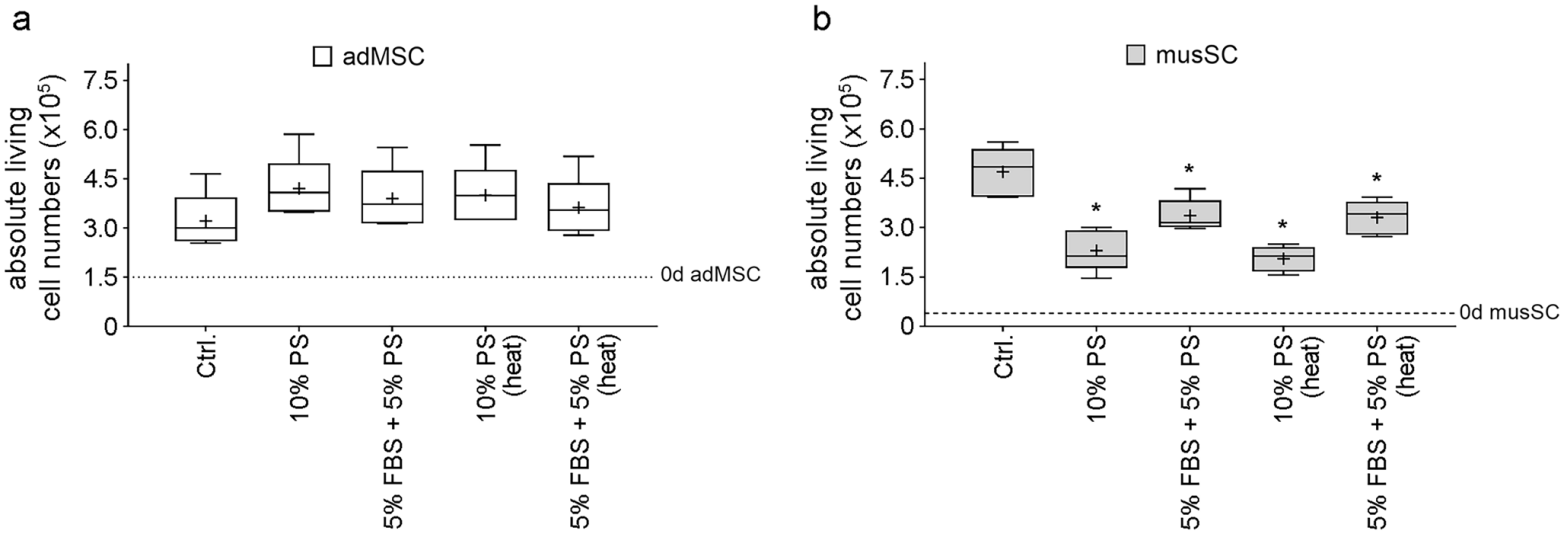
Absolute numbers of living cells after 72 h of cultivation using different serum supplementations. **a** Human adipose–derived mesenchymal stem/stromal cells (adMSC) and **b** porcine myoblasts derived from satellite cells (musSC) were cultured with 10% porcine serum (PS) or 5% PS + 5% fetal bovine serum (FBS), PS without or with heat inactivation (heat). Cell numbers were determined using the NucleoCounter^®^ NC-3000™. The numerical data are shown as boxplots, with medians, means (+), interquartile ranges, and minimum and maximum values (whiskers). The Shapiro-Wilk test indicates a Gaussian distribution; therefore, the statistical analysis was performed using an ANOVA test (ordinary one-way variance analysis) followed by Dunnett’s post hoc test (multiple comparison) with *P* (*) < 0.005, *n* = 5 with three technical replicates. Respective controls (Ctrl.): adMSC with 10% FBS, musSC with 10% FBS + 10% horse serum

The analysis of the cell numbers after 72 h of cultivation with complete or partial replacement of FBS by PS did not induce significant changes in adMSC compared with the FBS control cultures (*P* = 0.1811), indicating that the proliferative capacity of adMSC is not affected by the use of PS (Fig. 1a). As far as we know, there are no studies that examined the effects on cultivation of adMSC with PS. However, our results may complement the study by Tunaitis et al. (2011) who showed that human adMSC can be cultured successfully in sera supplements other than FBS, for example, with human (allogeneic) serum. In contrast to adMSC, musSC cell numbers were significantly affected by PS supplementation, regardless of the volume of PS addition (*P* < 0.0001). PS exposure resulted in reduced cell numbers compared to control cultures (*P* ≤ 0.049), indicating a reduction of musSC proliferation (Fig. 1b).

The viability of the cells was examined using an automated assay procedure after detachment of the cells, with adMSC generally showing higher viability than musSC. For the adMSC, the viability range from 98.15 to 98.99% was not considerably influenced by the addition of the differently processed sera. The viability of musSC showed a somewhat larger scatter and was between about 91.18% and 96.27%. Greater fluctuations within the viability for the musSC can be explained by the timing of the measurement, as the adMSC were measured directly after detachment, while the musSC first had to be transported for the measurement and were stored around 5 h in ice.

**Fig. 2:**
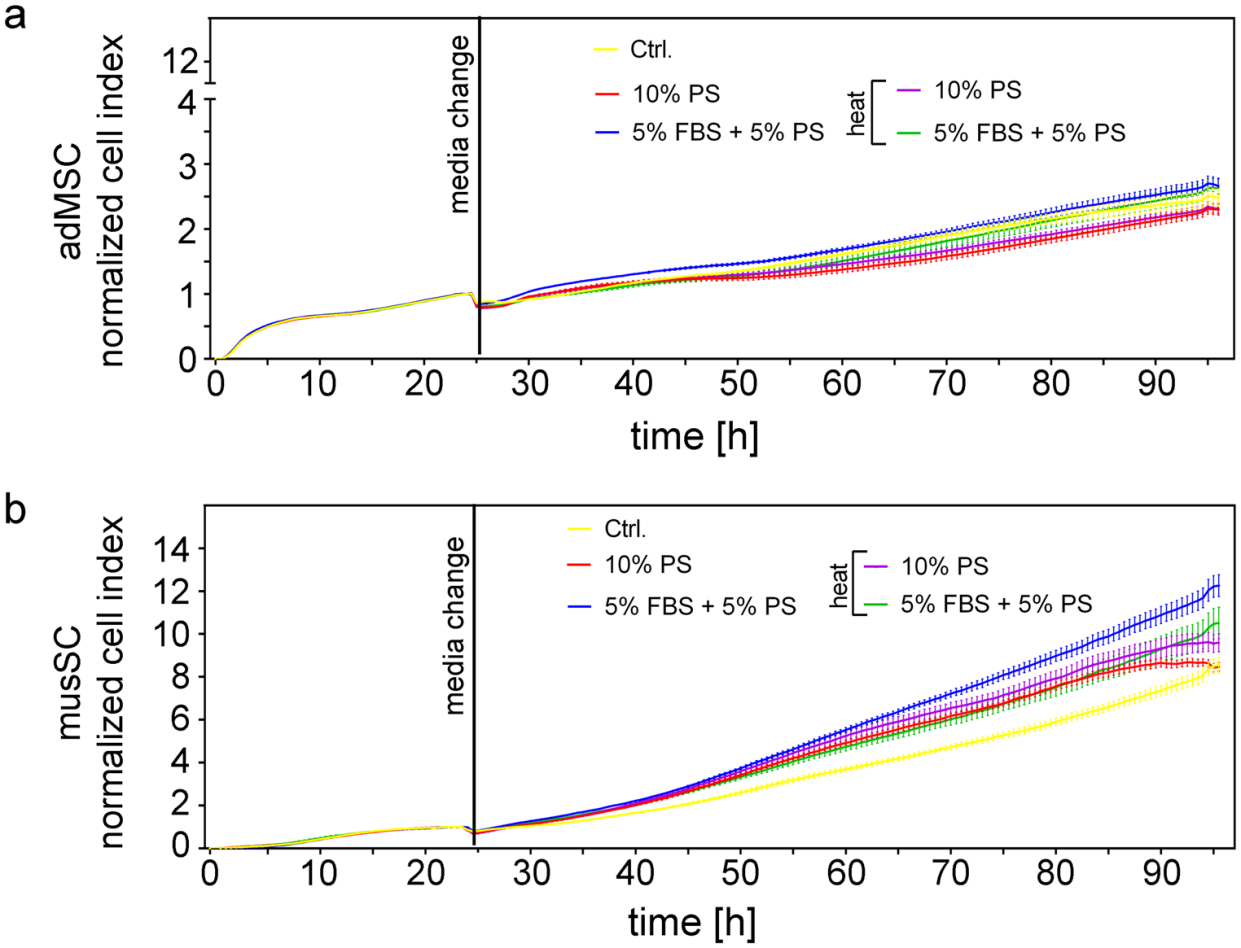
Real-time monitoring of **a** human adipose–derived mesenchymal stem/stromal cells (adMSC) and **b** porcine myoblasts derived from satellite cells (musSC) under the supplementation with different sera. Impedance measurements were recorded every 30 min over 96 h and are expressed as normalized cell index (nCI, means ± SEM) using the xCELLigence RTCA SP system. Cells were cultured for 24 h using growth medium with 10% fetal bovine serum (FBS) in the case of adMSC or with 10% FBS + 10% horse serum for porcine musSC. A medium change was then performed, during which the controls (Ctrl.) were cultured as described above for additional 72 h. Further adMSC and musSC were treated with 10% PS or 5% PS + 5% FBS, PS without or with heat inactivation (heat). *n* = 3 with three technical replicates

Impedance-based real-time monitoring of proliferation was performed over 96 h (72 h with different sera) by using the xCelligence RTCA SP system. This system generates a unitless cell index (CI) which was normalized to 1.0 at the time point of the addition of different sera (24 h of cultivation). The normalized cell index (nCI, mean ± standard error of the mean (SEM)) showed that adMSC (Fig. 2a) and musSC (Fig. 2b) were vital and proliferated when PS was used alone or in combination with 5% FBS. No PS-dependent changes in nCI were observed in adMSC (*P* = 0.192), with values ranging from 2.299 ± 0.085 to 2.662 ± 0.122 (Supplementary Table 3). The nCI of musSC, on the other hand, was increased by the partial replacement of 5% FBS by 5% PS compared with the control cultures, whether the PS was heat-inactivated or not (8.557 ± 0.355 vs. 10.520 ± 0.728 or 12.26 ± 0.504, *P* ≤ 0.018). Interestingly, the complete replacement of FBS by PS did not change the nCI of musSC (*P* ≥ 0.282). The steepness of the nCI curves described by the slope (mean ± SEM, [1/h]) was affected by PS in both cell types (*P* ≤ 0.022; Supplementary Table 3). However, in the case of adMSC, no significant changes were found when replacing FBS completely or partially by PS (*P* ≥ 0.087). In musSC, the slope through both 5% PS approaches was independent of heat inactivation (0.162 ± 0.007 or 0.135 ± 0.009 1/h with *P* ≤ 0.011) and for 10% heat-inactivated PS (0.138 ± 0.008 1/h with *P* = 0.0025) compared to control (0.105 ± 0.004 1/h) significantly increased. As can be seen in Fig. 2b, the musSC changed from exponential growth to the stationary phase about 65 h after the addition of 10% PS. In summary, the xCelligence data (nCI and slope) indicated generally faster proliferative behavior of musSC compared with adMSC, even when we seeded more adMSC (4500) than musSC (1500) per well. This is in line with studies reporting population doubling times of around 45 h for adMSC (Peng et al. 2008; Lotfy et al. 2014) and 20 h for musSC (Zammit et al. 2002; Metzger et al. 2021). This could be the cause for the reduced cell numbers and viability of musSC after 72 h as described above. On the other hand, the musSC of porcine origin seems to benefit from at least a low concentration of allogenic serum (5% PS) as shown via real-time monitoring, whereby the adMSC of human origin did not show any growth differences due to the use of (xenogenic) PS compared with standard FBS. This might be comparable to shorter doubling times described for human adMSC cultured in (allogenic) human serum instead of FBS (Hass et al. 2011). Heat inactivation of the PS apparently had no effect on the proliferative rate of either cell type (Fig. 1).

### Differentiation capacity

**Fig. 3:**
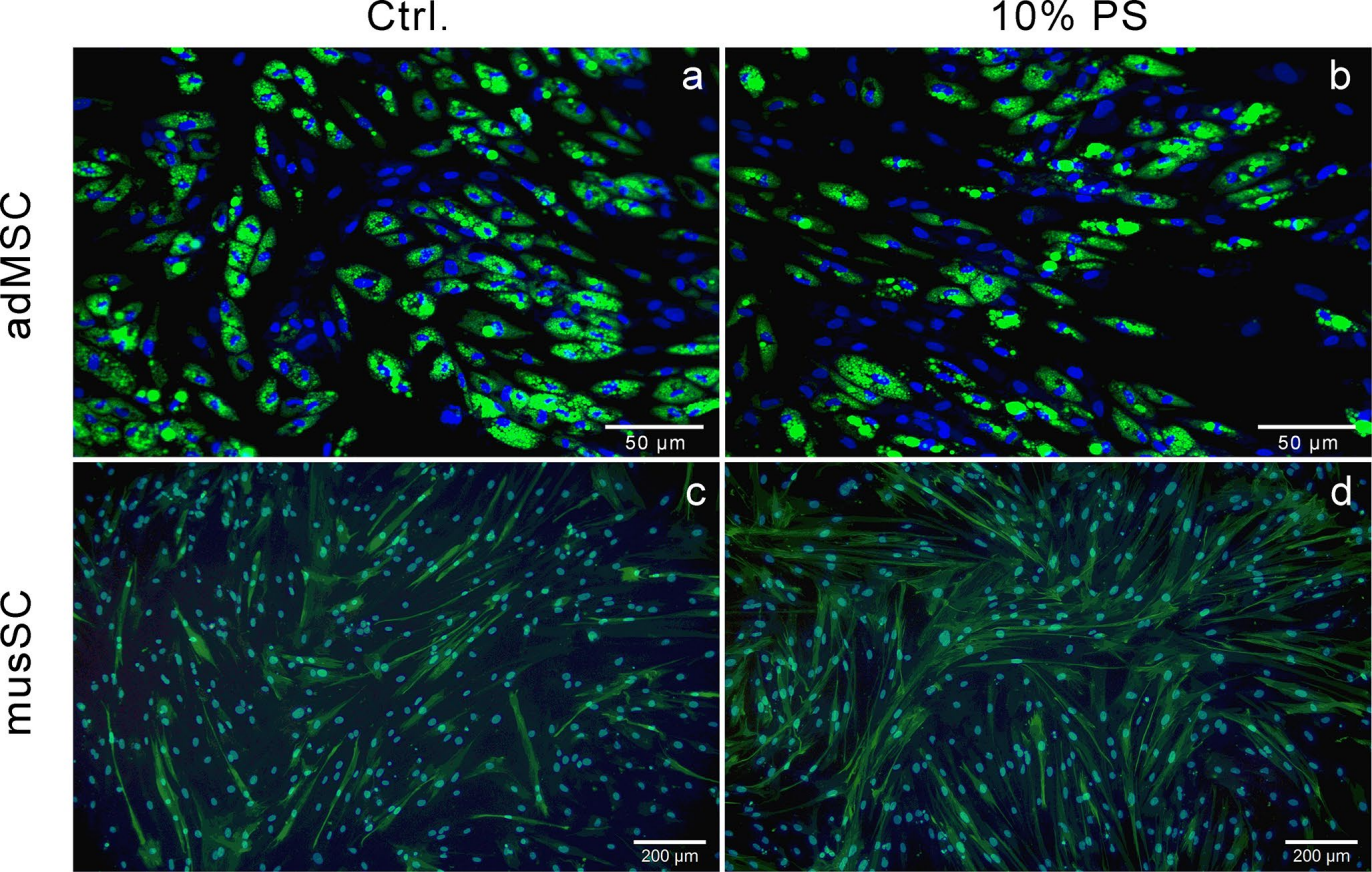
Detection of specific adipogenic (adipose-derived mesenchymal stem/stromal cells, adMSC) and myogenic (myoblasts derived from satellite cells, musSC) differentiation. Human adMSC were differentiated using **a** 10% fetal bovine serum (FBS, Ctrl.) or **b** 10% porcine serum (PS) without heat inactivation. As the standard myogenic differentiation protocol for musSC is under serum-free conditions, only pre-cultivation was done in the presence of **c** 10% FBS + 10% HS (Ctrl.) or **d** 10% PS (see the “Materials and methods” section for details). Differentiation was visualized by staining of lipid accumulation (adMSC) and by the presence of myotubes (musSC). Representative images taken with the Hermes WiScan System (for adMSC) or with a Leica DM 4000B microscope equipped with an Olympus DP74 camera (for musSC); scale bar, 50 µm (adMSC) or 200 µm (musSC); in green, lipid accumulation in adMSC or immunofluorescence of desmin in musSC; in blue, nuclear staining

Furthermore, we investigated whether the chosen adult stem cells were able to differentiate into the tissue-specific cell type (i.e., adipocytes and myotubes) by analyzing the effects of PS as a medium supplement compared with standard cultures with FBS (Fig. 3). The detection of specific differentiation markers (lipid accumulation for adipogenic differentiation of adMSC and myotube formation for myogenic differentiation of musSC) showed that specific cell differentiation was possible under the influence or following the influence of PS (Fig. 3b and d).

A different extent of lipid accumulation was observed microscopically, as cultivation with 10% PS showed a lower lipid accumulation compared with control (Fig. 3a and b, in green). A significant decrease in adipogenic differentiation was observed with the complete replacement of FBS by PS compared with control, whether the PS was heat-inactivated or not (Supplementary Fig. 1b, 2.574 ± 0.071 with *P* < 0.0001 and 2.848 ± 0.104 with *P* = 0.0002 vs. 3.616 ± 0.068). The fluorescent staining intensities of the partial approaches did not differ from the control and are comparable with the differentiation capacities of human adMSC in the presence of FBS. Studies using PS as a surrogate for FBS are sparse in the literature. However, Tunaitis et al. (2011) showed that MSC exhibit similar growth, differentiation, and immunophenotypic and proteomic properties in the presence of different serum supplements compared with FBS. Interestingly, we observed a simultaneous numeric increase in cell numbers under PS supplementation during adipogenic differentiation (Supplementary Fig. 1a). This is in contrast to previous studies showing that proliferation usually stagnates at the time of adipogenic differentiation (Wolff et al. 2022). Similarly, in porcine stromal vascular adipocytes, different sera supplements (including PS) stimulated initial proliferation and inhibited subsequent differentiation (Suryawan and Hu 1993). Although the increased cell number in our pilot experiment was not statistically significant, the sustained proliferation suggests a potential for optimization of adipogenic differentiation by PS addition.

As the standard myogenic differentiation protocol for musSC is under serum-free conditions, only pre-cultivation was done in the presence of FBS and HS (Fig. 3c) or PS (Fig. 3d) as mentioned above. Myogenic differentiation of musSC was analyzed by the degree of fusion, where a myotube was defined as two or more nuclei in a desmin-positive cell. In general, myoblasts from pigs achieved the lowest fusion rate of all farm animals examined by Bacquero-Perez et al. (2012), with approx. 41% of the cells fusing to form myotubes. Surprisingly, only the onset of differentiation in the presence of 10% PS resulted in a significantly higher fusion index compared with the control (58.1 ± 7.9% vs. 41.0 ± 1.3%, *P* = 0.014). The other PS-containing media did not affect the fusion degree (*P* ≥ 0.052), with a range from 47.1 ± 1.5 to 54.8 ± 1.1%. Doumit and Merkel (1992) found comparable fusion degrees of porcine myogenic satellite cells due to initial proliferation with 10% FBS or 10% PS and at least serum-reduced (2% FBS) differentiation. From our results, we expect that PS offers very good potential for optimizing our in vitro model in terms of myotube content. Furthermore, heat inactivation does not seem to play a significant role in the differentiation data either. Nevertheless, the aspect of heat inactivation and the associated inhibition of the donors’ mature complement system should be concretized in further studies, especially with regard to the composition of the PS. The extent to which our primary cells can also be isolated, preserved, and cryopreserved with PS should also be examined in future studies.

## Conclusion

Numerous efforts to develop a standardized cell culture supplement that would enable the general replacement of FBS have so far met with little success. Human platelet lysates from surplus donor blood are potentially suitable to replace FBS (van der Valk et al. 2018). However, this resource is limited, and the products obtained are very expensive (Gstraunthaler et al. 2015). Pig blood, as a by-product of slaughtering, represents a sufficiently available and sustainable resource for PS and could therefore receive more attention as an alternative for FBS replacement in in vitro models. Our results clearly show for the first time that the proliferation, cell viability, and differentiation capacity of primary human and porcine stem cells are maintained even when PS is added. The use of PS requires further research as the cultivability of each cell type depends on the species and/or tissue origin and should, therefore, be optimized for each in vitro model. In addition, PS derived from slaughter blood seems to be a promising basis for the development of new cell culture additives.

## Supplementary Information

Below is the link to the electronic supplementary material.

Supplementary file1 (PDF 581 KB)

## Data Availability

The authors declare that all data supporting the findings of this study are available within the article and Supplementary information or are available from corresponding authors upon reasonable request.
