# Supplementary material for: Potential of animal-welfare compliant and sustainably sourced serum from pig slaughter blood
Source: Cell Tissue Res. 2024 Jul 11;397(3):205–14. doi: 10.1007/s00441-024-03904-8 (PMC11371839; doi:10.1007/s00441-024-03904-8)
Supplement: Supplementary file 1 — Supplementary file1 (PDF 581 KB) [file 441_2024_3904_MOESM1_ESM.pdf]

Supplementary Information (SI)

Potential of animal-welfare compliant and sustainably sourced serum from pig slaughter blood

Olga Hahn[1], Kirsten Peters[1], Alexander Hartmann[2], Dirk Dannenberger[3] and Claudia Kalbe[3*]

[1]Institute for Cell Biology, University Medical Center Rostock, Rostock, Germany; [2]Institute of Clinical Chemistry and Laboratory Medicine, University Medical Center Rostock, Rostock, Germany; [3]Research Institute for Farm Animal Biology (FBN), Dummerstorf, Germany

*Corresponding author: kalbe@fbn-dummerstorf.de (C. Kalbe); Research Institute for Farm Animal Biology (FBN), Wilhelm-Stahl-Allee 2, D-18196 Dummerstorf, Germany

Human subjects

For the purpose of this study, all human donors gave written informed consent for tissue donation. In total, material from five patients (female) who had undergone tumescent liposuction was used. The mean age of the donors was 46.2 ± 3.1 years (between 43 and 50 years) with an average body mass index of 30.4 ± 3.0 kg/m^2.

Animals

The animals were obtained from the Experimental Animal Facility Pig of the Research Institute for Farm Animal Biology (FBN), Dummerstorf, Germany (70 days-old pigs) or from the Testing station of the Hybrid Pig Breeding Association North/East MV (fattening pigs) and were not part of animal experiments. Animal husbandry and slaughter followed the guidelines set by the Animal Care Committee the State of Mecklenburg-Western Pomerania, Germany, based on the German Law for Animal Protection.

For serum collection, ten female fattening pigs (171 ± 15 days of age) with a body weight of 124 ± 5 kg were killed using exsanguination after electro stunning.

For muscle cell culture, five female 70-day-old pigs with a body weight of 19.4 ± 1.1 kg were killed at the FBN Experimental abattoir using exsanguination after electro stunning. Tissue of the *M. rhomboideus* was dissected and washed in enriched phosphate buffered saline until the isolation procedure.

Media and buffer

adMSC (adipose tissue-derived mesenchymal stem/stromal cells)

Proliferation medium: DMEM containing 1% penicillin/streptomycin, 0.4% GlutaMax™ (all from Gibco by Life technologies, Darmstadt, Germany) with 10% FBS (PAN Biotech, Aidenbach, Germany, without heat inactivation)

Differentiation medium: proliferation medium containing 1 µM dexamethasone, 500 µM 3-isobutyl-1-methylxanthine, 200 µM indomethacin and 10 µM insulin (all from Sigma-Aldrich, Saint Louis, MO, USA)

musSC (skeletal muscle tissue-derived satellite cells)

Enriched phosphate buffered saline: 144 mM NaCl, 5.4 mM KCl, 25 mM glucose, 14 mM sucrose, 5 mM Na_2HPO_4, 1 µg/mL phenol red (all from Carl Roth, Karlsruhe, Germany), 50 IU/mL penicillin and 50 µg/mL streptomycin (both from Biochrom, Berlin, Germany), adjusted to pH 7.4 at 22°C

Proliferation medium: DMEM (PAN Biotech) supplemented with 0.2 M L-glutamine (Carl Roth), 100 IU/mL penicillin, 100 µg/mL streptomycin (both from Biochrom), 2.5 µg/mL amphotericin (Sigma-Aldrich), 10% FBS (Sigma-Aldrich, without heat inactivation) and 10% donor horse serum (HS; Sigma-Aldrich, without heat inactivation)

Proliferation medium 2: DMEM (PAN Biotech) supplemented with 0.2 M L-glutamine (Carl Roth), 100 IU/mL penicillin, 100 µg/mL streptomycin (both from Biochrom), 2.5 µg/mL amphotericin, 10% FBS (without heat inactivation) and 1 µM insulin (all from Sigma-Aldrich)

Differentiation medium: MEM (Biochrom) supplemented with 0.2 M L-glutamine (Sigma-Aldrich), 100 IU/mL penicillin, 100 µg/mL streptomycin (both from Biochrom), 2.5 µg/mL amphotericin, 1 µM insulin, 1 µM cytosine β-D-arabinofuranoside, 0.5 mg/mL bovine serum albumin, 0.1 nM dexamethasone, 0.5 µg/mL linoleic acid and 100 µg/mL transferrin (bovine holoform, all from Sigma-Aldrich)

Supplementary Table 1: Amino acid composition of the two routinely used fetal bovine sera (FBS) and the sustainable pig serum (PS) derived from slaughter blood

**Amino acids** (µM)	**FBS** (PAN Biotech, Lot No.: P160406)	**FBS** (Sigma-Aldrich, BCBW7811)	**PS**
**Proteinogenic amino acids**			
Ala (alanine)	1117.5	1017.1	707.8
Arg (arginine)	184.7	32.3	80.4
Asn (asparagine)	82.5	8.2	16.0
Asp (aspartic acid)	186.6	90.5	10.2
Cys (cysteine)	63.2	19.9	54.2
Gln (glutamine)	315.5	370.8	331.9
Glu (glutamic acid)	1343.1	828.6	405.2
Gly (glycine)	714.5	566.1	1022.3
His (histidine)	167.3	71.7	83.6
Ile (isoleucine)	535.6	121.3	132.8
Leu (leucine)	625.2	203.2	244.4
Lys (lysine)	557.2	174.2	238.6
Met (methionine)	122.2	18.6	33.1
Phe (phenylalanine)	324.2	128.8	95.3
Pro (proline)	271.8	102.3	437.2
Ser (serine)	443.6	234.8	164.3
Thr (threonine)	536.6	140.4	184.3
Trp (tryptophan)	95.8	46.4	64.0
Tyr (tyrosine)	238.0	85.8	68.6
Val (valine)	679.0	304.6	422.8

**Non-proteinogenic amino acids**			
α-AAA (α-aminoadipine acid)	40.3	31.8	111.8
α-ABA (α-aminobutyric acid)	52.2	40.8	18.1
anserine	1.9	0.8	0.4
β-alanine	30.5	37.9	14.4
carnosine	12.2	15.5	4.2
citrulline	73.1	79.0	66.2
GABA (γ-aminobutyric acid)	9.2	9.6	3.3
hydroxyproline (Hyp)	71.4	85.0	57.2
iso-ABA (iso-aminobutyric acid)	3.7	2.1	0.4
1-Met-His (1-methyl-histidine)	12.1	13.6	2.6
3-Met-His (3-methyl-histidine)	83.3	49.6	8.5
ornithine	260.5	218.3	225.0
taurine	102.4	148.1	267.4

Supplementary Table 2: Fatty acid concentration of the two routinely used fetal bovine sera (FBS) and the sustainable pig serum (PS) derived from slaughter blood

**Fatty acids** (µg per g serum)	**FBS** (PAN Biotech, Lot No.: P160406)	**FBS** (Sigma-Aldrich, BCBW7811)	**PS**
**Saturated fatty acids**			
C8:0	0.12	0.00	0.06
C10:0	0.50	0.08	0.84
C12:0	0.98	0.82	1.09
C13:0	0.12	0.03	0.08
C14:0	12.27	6.84	9.13
C15:0	7.76	3.53	3.06
C16:0	158.90	88.51	266.19
C17:0	6.50	3.72	8.66
C18:0	126.91	43.01	177.05
C20:0	1.32	1.56	1.12
C21:0	0.56	0.79	0.46
C22:0	0.68	2.82	9.01
C23:0	1.54	1.98	1.42
C24:0	4.21	4.46	2.87
C26:0	2.11	2.83	1.66
**Mono-unsaturated fatty acids**			
C14:1*cis*-9	0.43	0.00	0.04
C16:1*cis*-9	22.94	10.62	14.63
C17:1*cis*-9	7.35	0.00	0.19
C18:1*trans*-9	2.61	0.38	4.20

C18:1*trans*-11	8.73	0.48	0.59
C18:1*cis*-9	150.16	69.89	286.72
C18:1*cis*-11	22.36	19.32	27.55
C20:1*cis*-11	0.86	0.93	2.77
C22:1*cis*-13	0.27	0.00	0.29
**Poly-unsaturated fatty acids**			
C18:2*trans*-9,*trans*-12	0.56	0.38	0.15
C18:2n-6	133.35	15.85	481.78
C18:3n-6	4.15	0.77	4.49
C18:3n-3	103.69	1.17	15.68
C18:2cis-9,trans-11	3.03	0.57	0.68
C18:4n-3	2.81	0.45	0.15
C20:2n-6	0.93	0.39	4.49
C20:3 n-9	3.06	1.33	2.13
C20:3n-6	15.02	6.33	7.68
C20:3n-3	0.40	0.00	0.87
C20:4n-6	43.38	19.21	125.69
C22:2n-6	0.35	0.00	0.17
C20:5n-3	27.02	2.90	4.77
C22:4n-6	2.35	0.84	7.68
C22:5n-6	1.80	0.00	1.07
C22:5n-3	14.80	5.39	14.16
C22:6n-3	14.68	13.01	5.74

Supplementary Table 3: Serum-dependent normalized cell indices (nCI) and slopes of human adMSC and porcine musSC over a period of 72 hours

Impedance measurements (xCELLigence, ACEA Biosciences Inc) were recorded every 30 min for up to 96 hours and results were expressed as nCI. The nCI and slope values provided by the system (Software RTCA, Version 1.2.1.) between 24 and 96 hours were used for our statistical analyses. Numerical data are presented as means with SEM. When the Shapiro-Wilk test indicated a non-Gaussian distribution, statistical analysis was performed using the Kruskal-Wallis test, followed by a Dunn multiple comparison test with *P < 0.05 compared to the control (Ctrl.). For data with a Gaussian distribution, an ANOVA test (Ordinary one-way analysis of variance) was performed, followed by a Dunnett post hoc test (multiple comparison) with *P < 0.05 compared to control. The experiments were performed with three independent adMSC or musSC preparations and three technical replicates. FBS: fetal bovine serum; PS: porcine serum

				**with heat**		
	**Ctrl.**	**10% PS**	**5% FBS + 5% PS**	**10% PS**	**5% FBS + 5% PS**	**P value**
**adMSC**						
**nCI**	2.487 ± 0.135	2.299 ± 0.085	2.662 ± 0.122	2.316 ± 0.077	2.628 ± 0.144	0.1921
**slope [1/h]**	0.024 ± 0.002	0.019± 0.001	0.025 ± 0.002	0.020 ± 0.001	0.025 ± 0.002	0.0215
**musSC**						
**nCI**	8.557 ± 0.355	8.465 ± 0.200	12.26*± 0.504	9.602 ± 0.424	10.52* ± 0.728	<0.0001
**slope [1/h]**	0.105 ± 0.004	0.126 ± 0.003	0.162*± 0.007	0.138*± 0.008	0.135* ± 0.009	<0.0001

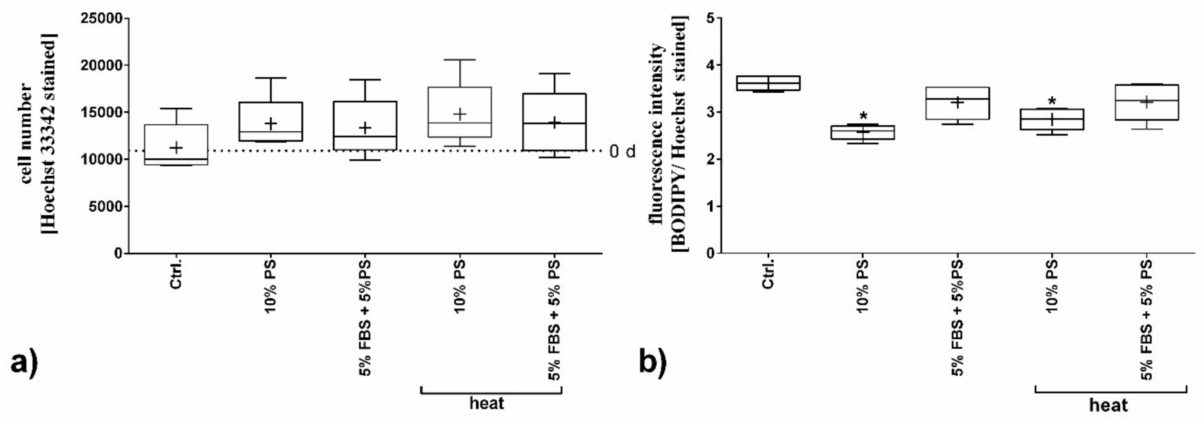

Supplementary Fig. 1 Quantification of **(a)** the cell number and **(b)** bodipy-fluorescence intensity after specific adipogenic differentiation of adMSC

Cells were cultured for up to 14 days with adipogenic stimulated medium with 10% fetal bovine serum (FBS) and with the following serum supplements: 10% porcine serum (PS), 5% FBS + 5% PS without or with heat inactivation. Cell numbers were analyzed by quantification of cell nuclei after staining with Hoechst 33342 using the Hermes WiScan and the Athena Software (IDEA Bio.Medical, Israel). Lipid accumulation was determined by bodipy-fluorescence intensity (values were normalized to respective cell numbers). Numerical data are presented as box plots with medians, means (+), interquartile ranges, and minimum/maximum values. The statistical analysis was performed using the Two-Way variance analysis test ANOVA followed by Dunnett's multiple comparison test compared to the respective control (Ctrl. 10% FBS; *P < 0.005). The experiment was performed with five independent adMSC preparations with three technical replicates.
